# Screening performances of an 8-item UPSIT Italian version in the diagnosis of Parkinson’s disease

**DOI:** 10.1007/s10072-022-06457-2

**Published:** 2022-11-19

**Authors:** Annamaria Landolfi, Marina Picillo, Maria Teresa Pellecchia, Jacopo Troisi, Marianna Amboni, Paolo Barone, Roberto Erro

**Affiliations:** 1 Department of Medicine, Surgery and Dentistry “Scuola Medica Salernitana”, Neuroscience Section, University of Salerno, Via Allende 43, 84081 Baronissi, SA Italy; 2 Theoreo srl, Via degli Ulivi 3, 84090 Montecorvino Pugliano, Italy; 3 Department of Chemistry and Biology “A.Zambelli”, University of Salerno, Fisciano, Italy; 4 Istituto di Diagnosi e Cura Hermitage-Capodimonte, Naples, Italy

**Keywords:** University of Pennsylvania Smell Identification Test, Smell impairment, Parkinson’s disease, Machine learning

## Abstract

Hyposmia is a common finding in Parkinson’s disease (PD) and is usually tested through the University of Pennsylvania Smell Identification Test (UPSIT). The aim of our study is to provide a briefer version of the Italian-adapted UPSIT test, able to discriminate between PD patients and healthy subjects (HS). By means of several univariate and multivariate (machine-learning-based) statistical approaches, we selected 8 items by which we trained a partial-least-square discriminant analysis (PLS-DA) and a decision tree (DT) model: class predictions of both models performed better with the 8-item version when compared to the 40-item version. An area under the receiver operating characteristic (AUC-ROC) curve built with the selected 8 odors showed the best performance (sensitivity 86.8%, specificity 82%) in predicting the PD condition at a cut-off point of ≤ 6. These performances were higher than those previously calculated for the 40-item UPSIT test (sensitivity 82% and specificity 88.2 % with a cut-off point of ≤ 21). Qualitatively, our selection contains one odor (i.e., apple) which is Italian-specific, supporting the need for cultural adaptation of smell testing; on the other hand, some of the selected best discriminating odors are in common with existing brief smell test versions validated on PD patients of other cultures, supporting the view that disease-specific odor patterns may exist and deserve a further evaluation.

**Supplementary Information:**

The online version contains supplementary material available at 10.1007/s10072-022-06457-2.

## Introduction

Hyposmia represents one of the most frequent non-motor symptoms in Parkinson’s disease (PD), affecting more than 90% of PD patients [1, 2]. It can be detected very early in the course of the disease, often before motor symptoms start [1]. For this reason, olfaction dysfunction has been proven to be a reliable, early predictive marker for PD, with smell evaluation testing being as sensitive as the gold standard instrumental investigation, i.e., single-photon emission computed tomography (SPECT) for dopamine transporter (DaT) study [3]. The University of Pennsylvania Smell Identification Test (UPSIT) is the most employed tool to detect olfactory dysfunction in PD patients, in both clinical and research settings [4]. It consists of 40 microencapsulated odorants which are released by scratching standardized odor-impregnated strips [5]. Its use has been validated across different cohorts worldwide [6–9], with cultural item adaptation. We have previously developed the Italian version of the UPSIT in which some odors that are virtually unknown to Italian subjects, such as cheddar cheese, gingerbread, and turpentine were replaced by other odors, validating it in healthy subjects [10] and in PD [7].

A number of briefer smell tests have been subsequently developed (for example, the brief smell identification test (B-SIT) [11] and its version adapted to PD, namely the BSIT-B [12]), with the aim of abbreviating and, thus, optimizing smell evaluation in the routine clinical practice. As reviewed by Morley et al., [13] they reached fairly good diagnostic performances when compared to the UPSIT test. However, not all of them have been validated on the PD population. Therefore, abbreviated versions of the UPSIT have been tested in several studies, with reasonable predictive performance for PD [13, 14]. As for the full-length, 40-item version of the UPSIT, cross-cultural adaptation of brief versions is necessary. Therefore, the aim of the current work is to develop an abbreviated version of the Italian-adapted UPSIT test. To this aim, we employed several univariate and multivariate (machine learning-based) statistical approaches in order to select the best items of the 40-item UPSIT in discriminating PD patients from healthy subjects (HS).

## Materials and methods

### Study population

The current work stems from a secondary analysis of data from our previous study validating the culturally adapted version of the 40-item UPSIT smell test for the Italian population [7], with enrollment performed prior to the Covid-19 pandemic outbreak, thus excluding the possibility of SARS-CoV-2 infection-related hyposmia. In brief, the study subjects consisted of PD patients, as diagnosed according to the UK Brain Bank Criteria [15], compared to HS. Exclusion criteria for patients and HS were as follows: dementia, active upper respiratory tract inflammation, history of diabetes, nose surgery, or head trauma. The exclusion criterion for HS was a history of neurologic and/or psychiatric disease. All subjects underwent olfaction evaluation under medical supervision through the Italian version of the 40-item UPSIT.

### Statistical Analysis

#### Univariate analysis

Individual responses to each of the 40 items were recorded as correct or incorrect. In order to calculate the performance of an UPSIT odor subset, as predictive of PD diagnosis, we computed the discriminatory power of each odor in differentiating PD from HS using different statistical methods. First, we assessed the diagnostic performance of each item by calculating the correct/incorrect answer ratio for each class and *p*-values using the Fisher *χ*^2^ test. Secondly, we calculated diagnostic odds ratios for each item. Thirdly, we calculated the area under the receiver operating characteristic (AUC-ROC) curve for each item using the statistical software SPSS ver.26 (IBM Corp., Armonk, NY, USA).

#### Machine learning algorithms: logistic regression and linear discriminant analysis

The discriminatory power of each odor in differentiating PD from HS was also evaluated using multivariate (machine-learning-based) statistical models: logistic regression (LR) and linear discriminant analysis (LDA) [16, 17]. A Full explanation of the mathematical bases of these two models can be found in Supplementary Information (Online resource).

LR was performed using the MetaboAnalystR Package [18]. LDA was performed using the corresponding operator from RapidMiner v. 9.10 [19]. This operator needed no setting, so it was used as it is. UPSIT items were ranked according to their weight in discriminating the two classes.

#### UPSIT item selection

For each statistical model, we selected the top 12 items with the best discriminating performance. The UpSetR plot, which is a diagram to visualize intersections of multiple sets [20], was employed to aggregate these items in a combination matrix in order to show their simultaneous selection by several statistical approaches. The items which resulted as being selected by at least 4 out of 5 models were used to train a partial least square-discriminant analysis (PLS-DA) model [21], which is a supervised method that uses multivariate regression techniques to extract, via linear combinations of original variables (*X*, in our case, the best-selected odor items), the information that can predict class membership (*Y*, in our case, PD vs. HS). This PLS-DA model has then been submitted to a cross-validation process, by means of which we constructed a confusion matrix to synthetize correct and incorrect attributions. This was accompanied by diagnostic performance (in terms of sensitivity, specificity, negative and positive likelihood ratio, negative and positive predictive value, accuracy, AUCROC) calculation of item combinations (MetaboAnalystR Package) [18]. Also, a decision tree (DT) [22] was built with the same best-discriminating items and the related diagnostic performances were calculated. (RapidMiner 9.10). The concept behind DT functioning is explained in Supplementary Information (Online resource). DT was subjected to a cross-validation process similar to PLS-DA.

Finally, an AUCROC curve was build using the same best-discriminating items and cut-off points for assigning subjects to the PD group were calculated (SPSS ver. 26), the best threshold being evaluated using the Youden index [23].

## Results

### Demographic and clinical features of the studied populations

UPSIT examinations were obtained from a population of 68 PD patients and 61 healthy subjects [7]. The two populations were homogeneous in terms of age (61.8 ± 8.5 vs. 59.5 ± 8.5 for PD and subjects, respectively, *p* = 0.1), sex distribution (men/women distribution of 58.8%/41.2% for PD and 44.3%/55.7% for HS, *p* = 0.6), and smoking status (14.7% of PD smokers vs. 16.4% of HS smokers, *p* = 0.4). In the PD group, the mean Unified PD Rating Scale, motor sub-scale (UPDRSIII) value was 14.1 ± 6.4, and the mean Hoehn and Yahr stage was 1.6 ± 0.4. As expected, the mean 40-item UPSIT score was significantly lower in PD than in HS (16.8 ± 4.9 vs. 26.6 ± 507, *p* < 0.001).

### Odor selection

**Fig. 1:**
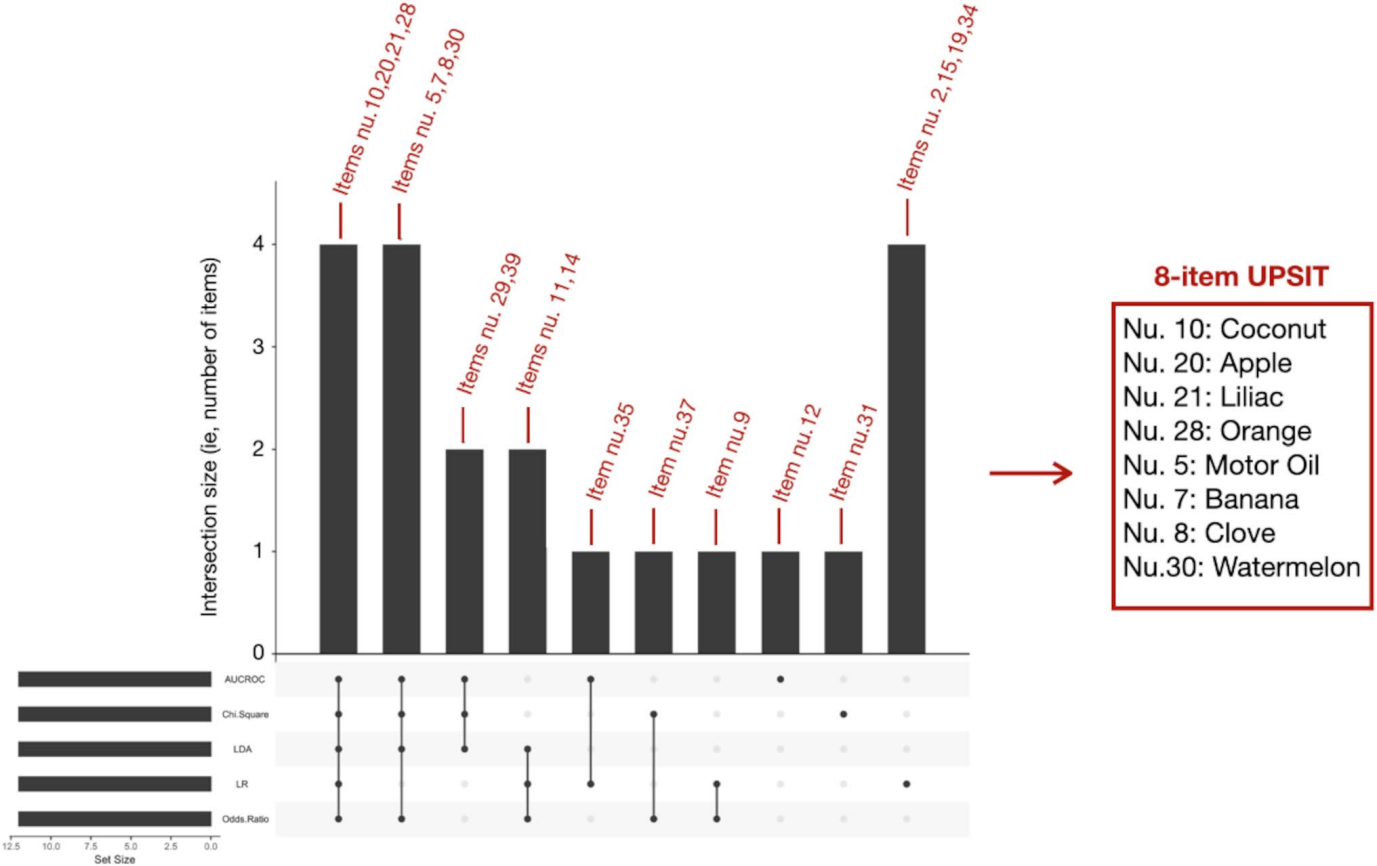
UpSetR plot showing odors selected across the different statistical models. The red numbers represent the items (odors), as they are numbered in the UPSIT test, selected by the different statistical models. Odors selected by at least 4 statistical models are shown on the right, being the final 8-item UPSIT. Column height depends on how many items are selected by the statistical models shown at the bottom (Abbreviations: LR = logistic regression, LDA = linear discriminant analysis, AUC-ROC= area under the receiver operating characteristic curve). The set size (i.e., the black bars on the left) represents the number of items chosen for each statistical method (in our case, 12)

**Fig. 2:**
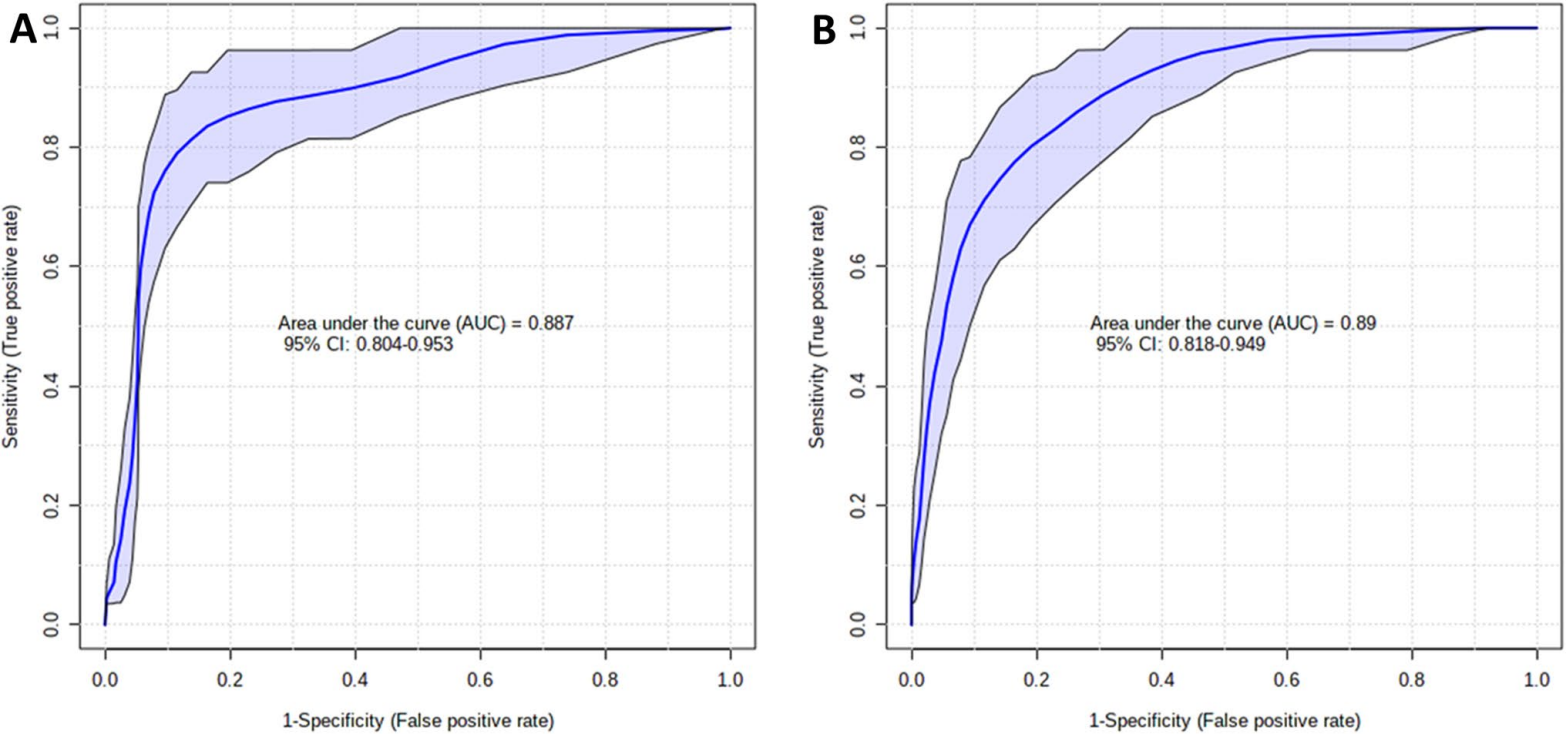
PLS-DA-built AUC-ROC curves comparing 8-item (**A**) and 40-item (**B**) AUCs

Five different statistical ranking strategies were used in order to select the best discriminating odors. Supplementary Table 1a shows the top 12 items with the best discriminating performance for each statistical method. Their occurrence or co-occurrence in one or more feature selection strategies was evaluated by means of an UpSetR plot (see Fig.1). As a result, 4 odors (namely, coconut, apple, lilac, orange) were selected by 5 models, 4 odors (namely, motor oil, banana, clove, watermelon) were selected by 4 models, 4 odors (namely, onion, talc, walnut, rose) were selected by 3 models, and 9 odors (chewing-gum, leather, fruit juice, cinnamon, chocolate, diluent, pine, grape, soap) were selected by only 1 model. This resulted in 8 items selected by at least 4 statistical models, (see Fig. 1 and items in bold, supplementary Table 1a) which were used to build a PLS-DA model for prediction performance calculation. The 8-item performances were compared to the PLS-DA-based prediction performances of the 40-item odor set. As shown in Table 1, the 8-item odor subset outperformed the 40-item odor set in terms of sensitivity (82% vs. 79%), specificity (92% vs. 85%), positive likelihood ratio (10:05 vs. 5,38), negative likelihood ratio (0:19 v. 0:24), positive predictive value (92% vs. 86%), negative predictive value (82% vs. 79%), and accuracy (86% vs. 82%). Figure 2 compares PLS-DA-based AUCROC curves for the 8-item and the 40-item set (0.887 vs. 0.89 respectively). Two DTs were built employing and comparing the 8 best discriminating items and the whole set of 40 items. They are shown in supplementary Fig. 1 and 2 (Online resource). In both trees, item 7 (i.e., banana) represents the starting node, and item 8 (i.e., clove) represents the immediately subsequent node with a huge relevance if correctly identified, in PD exclusion. As secondary nodes, items 10 (i.e., coconut) and 30 (i.e., watermelon) are shared by both trees in having a certain relevance in discriminating PD from healthy subjects. The diagnostic performances of the two DTs are shown in Table 1. They are lower than those obtained with PLS-DA but again, the 8-item subset performs better than the whole odor set of 40 items with respect to sensitivity (72% vs. 65%), specificity (85% vs. 80%), positive likelihood ratio (4:88 vs. 3:29), negative likelihood ratio (0:33 vs. 0:44), positive predictive value (84% vs. 79%), negative predictive value (73% vs. 67%), and accuracy (78.3% vs. 72.1%).

**Table 1:** PLS-DA-based and DT-based predictive performances of the 8-item odor subset compared to the whole odor set (values are shown as estimate ± standard error)

	PLS-DA based		DT based	
	8-item subset	40-item set	8-item subset	40-item set
Sensitivity	0.82 ± 0.05	0.79 ± 0.05	0.72 ± 0.05	0.65 ± 0.06
Specificity	0.92 ± 0.04	0.85 ± 0.05	0.85 ± 0.05	0.80 ± 0.05
Positive likelihood ratio	10.05	5.38	4.88	3.29
Negative likelihood ratio	0.19	0.24	0.33	0.44
Negative predictive value	0.82 ± 0.05	0.79 ± 0.05	0.73 ± 0.05	0.67 ± 0.05
Positive predictive value	0.92 ± 0.04	0.86 ± 0.04	0.84 ± 0.05	0.79 ± 0.05
Accuracy	0.868	0.822	0.783	0.721

Finally, the AUCROC curve built with the selected 8 odors (coconut, apple, lilac, orange, motor oil, banana, clove, watermelon) showed the best performance (sensitivity 86.8%, specificity 82%) in predicting the PD condition at a cut-off point of ≤ 6. These performances were higher than those calculated for the 40-item UPSIT test (sensitivity 82% and specificity 88.2 % with a cut-off point of ≤ 21) [7].

## Discussion

In the present work, by means of several univariate and multivariate (machine learning) statistical algorithms, we selected the 8 best UPSIT items in discriminating PD patients from HS. Machine learning supervised approaches (in our case, PLS-DA and DT) were also employed to train and cross-validate models for PD vs. HS class prediction. These two statistical algorithms showed a better diagnostic performance when dealing with the selected 8 items than when dealing with the whole set of 40 odors. This is intrinsically related to machine learning behavior. Indeed, data dimension reduction, which means that the number of features (in our case, the UPSIT items) is limited with respect to the number of observations (in our case, subjects), makes the training of classifiers more effective and decreases overfitting occurrence [24]. Accordingly, one of the principal concepts (informally known as “garbage in garbage out”) of computer science is that the better the quality of input data, the better the output is [25].

In addition, a comparison of our short test with the leader short smell identification test among those validated for PD (i.e., the 12-item BSIT-B, as evaluated by Joseph et al. [14]), showed, at the best sensitivity/specificity combination, a much better specificity with a quite negligible difference in sensitivity (sensitivity/specificity of 86.8/82% with a cut-off point of ≤ 6 vs. 96.5/51.8% with a cut-off point of ≤ 9, respectively), which is valuable aiming to screen for PD among subjects who might have lower smell performance due to other reasons, including aging.

As highlighted in the introductory section, many efforts in selecting UPSIT odor subsets and in testing their ability in identifying PD subjects have been performed in other cultures. Our 8-item selection shares a few items with previously published works. Indeed, “orange” and “clove” were also selected by Joseph et al. [14], who chose 2 “winning” 7-items subsets of the UPSIT from all the possible combinations containing 1–7 smells. Moreover, “banana” and “motor oil” were also selected by Morley et al. [13], who chose the 12 best discriminating smells as selected from the UPSIT using a combination of different statistical ranking strategies. Furthermore, common items have been also identified with the BSIT-B test, namely “banana,” “clove,” and “coconut”[12]. The item “banana” was also identified as one of the best discriminating items between PD and HS by Bohnen et al. [26] and in a shorter 5-item version of the B-SIT test by Double et al. [27]. Also, this item was also selected, via the random forest machine-learning approach, as one of the three best PD vs. HS discriminating odors from the 16-item “Sniffin’ Sticks” test [28]. This aspect of common patterns in odor identification has raised the argument that there may be a selective hyposmia in PD [12], yet with conflicting results [29]. Indeed, “clove” was also selected in two studies aimed at finding UPSIT subsets able to predict Alzheimer’s disease, although the two selected subsets were not sufficiently consistent with each other [30, 31]. It should be also acknowledged that a qualitative comparison between previously developed abbreviated versions of the UPSIT is not entirely possible because of cultural adaptations: indeed, one of the items selected by our statistical model (i.e., “apple”), is Italian-specific and, as such, not present in the original UPSIT version; likewise, other odor sub-selections described in Morley et al. [13] and Joseph et al. [14] contain culturally specific items (i.e., items that have been substituted in the Italian UPSIT version). These are “root beer” and “gingerbread” in Joseph et al.[14] and “turpentine” in Morley et al. [13]. This raises concern about the fact that odor identification patterns in the available published cohorts might be, at least partially, dependent on cultural issues. This aspect might also explain the fact that the results obtained in the discovery cohort were not entirely confirmed when reassessed in an independent cohort [13]. More in general, however, the PD-predictive ability of 4 “cross-cultural” odors identified by Joseph et al. [14], was confirmed after validation on an independent, even though geographically linked, PD cohort [32], supporting the concept of a specific quality of smell dysfunction in PD.

A few studies have tried to look for distinctive patterns of smell impairment among hyposmic patients of various causes. A study by Hähner et al. [33] investigated PD patients in comparison to patients with smell loss from other causes, by using the 16-item “Sniffin’ Sticks” test; they found no differences in odor identification thresholds and patterns between the two populations. On the contrary, in a Japanese cohort of PD and post-viral hyposmic patients evaluated with the Open Essence test, two odors (namely, menthol and Indian Ink) were found to accurately differentiate the two cohorts of hyposmic patients [34]. These heterogenous findings remark on the need of obtaining culturally adapted versions of specific smell evaluation tests, the performances of which should be evaluated according to the specific research purpose. These tests might in fact be proven useful to discriminate between PD and hyposmic patients due to other reasons and/or to differentiate PD from other non-degenerative parkinsonian syndromes [35].

## Conclusion

We have here presented an abbreviated 8-item UPSIT with a high accuracy in differentiating PD patients from healthy subjects, which makes smell evaluation much less time-consuming and feasible in routine clinical practice. We further showed that machine-learning-based odor selection is able to optimize this process, outperforming diagnostic performances of the full-length 40-item UPSIT. In this regard, however, we acknowledge a limitation of our study in the lack of validation on an independent PD cohort. Further studies are also warranted to explore whether the selected items are PD-specific by evaluating other populations affected by hyposmia due to other reasons and whether it accurately discriminates PD from other parkinsonian syndromes.

## Supplementary Information

Supplementary Fig. 1 Decision tree built with the best-discriminating 8 UPSIT items. Grey boxes represent nodes defined by item (odor) number. Branches are defined by the given answer to each item (0=incorrect; 1=correct). Red bars represent PD patients; blue bars represent HS; bar thickness represents the number of patients defined by each node; this number is also shoved within each box as “n:” For each bar, the red and the blue relative width represents the percentage of PD patients and HS identified by each node. (PNG 336 kb)

High Resolution Image (TIF 2715 kb)

Supplementary Fig. 2 Decision tree built with the whole UPSIT items. Grey boxes represent nodes defined by item (odor) number. Branches are defined by the given answer to each item (0=incorrect; 1=correct). Red bars represent PD patients; blue bars represent HS; bar thickness represents the number of patients defined by each node; this number is also shoved within each box as “n:” For each bar, the red and the blue relative width represents the percentage of PD patients and HS identified by each node. (PNG 337 kb)

High Resolution Image (TIF 2713 kb)

Supplementary file 1 (PDF 75 kb)
