# Supplementary figures and images for: Screening performances of an 8-item UPSIT Italian version in the diagnosis of Parkinson’s disease
Source: Neurol Sci. 2022 Nov 19;44(3):889–95. doi: 10.1007/s10072-022-06457-2 (PMC9676802; doi:10.1007/s10072-022-06457-2)

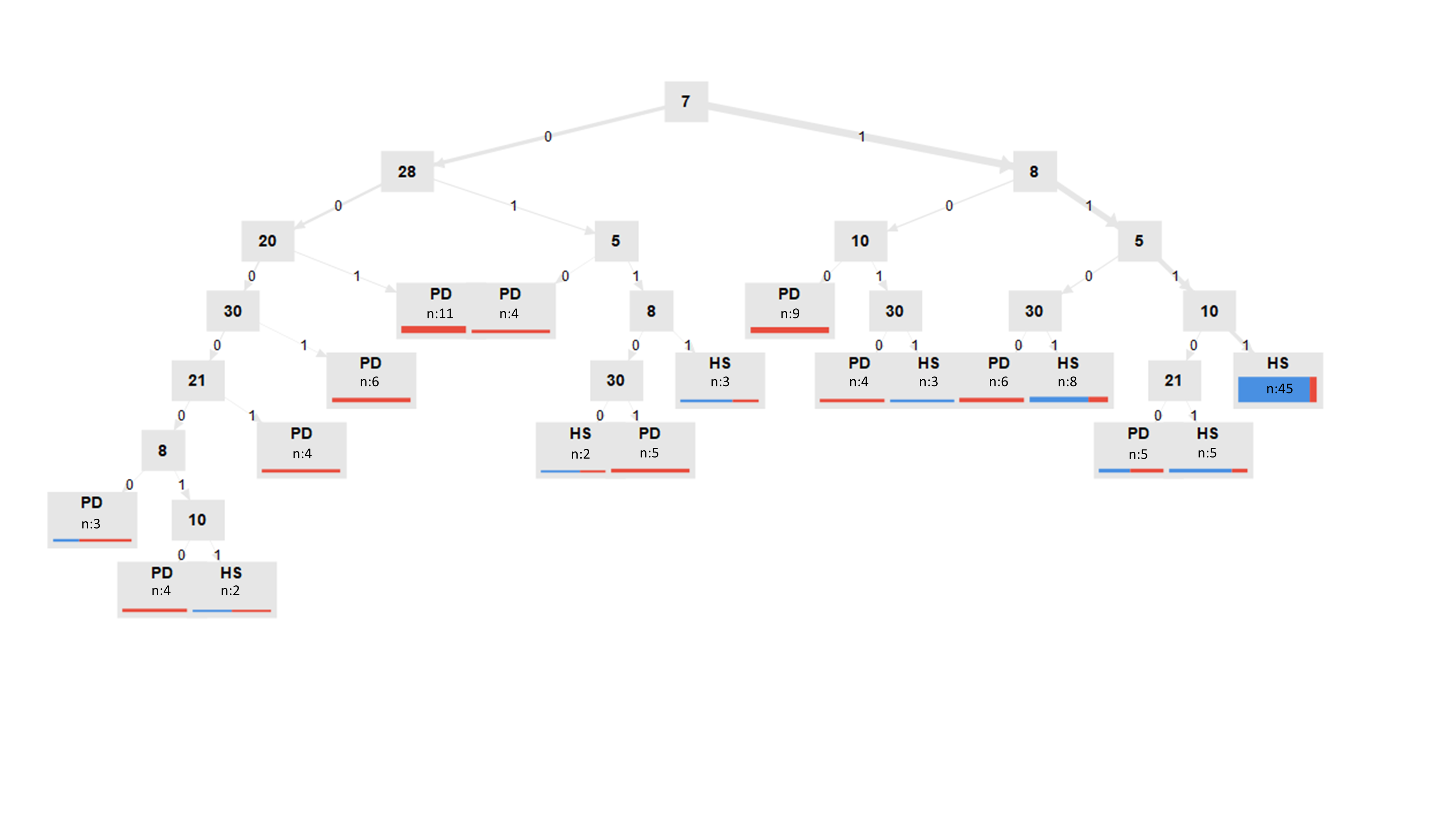

Supplement: Supplementary file 1 — Decision tree built with the best-discriminating 8 UPSIT items. Grey boxes represent nodes defined by item (odor) number. Branches are defined by the given answer to each item (0=incorrect; 1=correct). Red bars represent PD patients; blue bars represent HS; bar thickness represents the number of patients defined by each node; this number is also shoved within each box as “n:” For each bar, the red and the blue relative width represents the percentage of PD patients and HS identified by each node. (PNG 336 kb) [file 10072_2022_6457_Fig3_ESM.png]

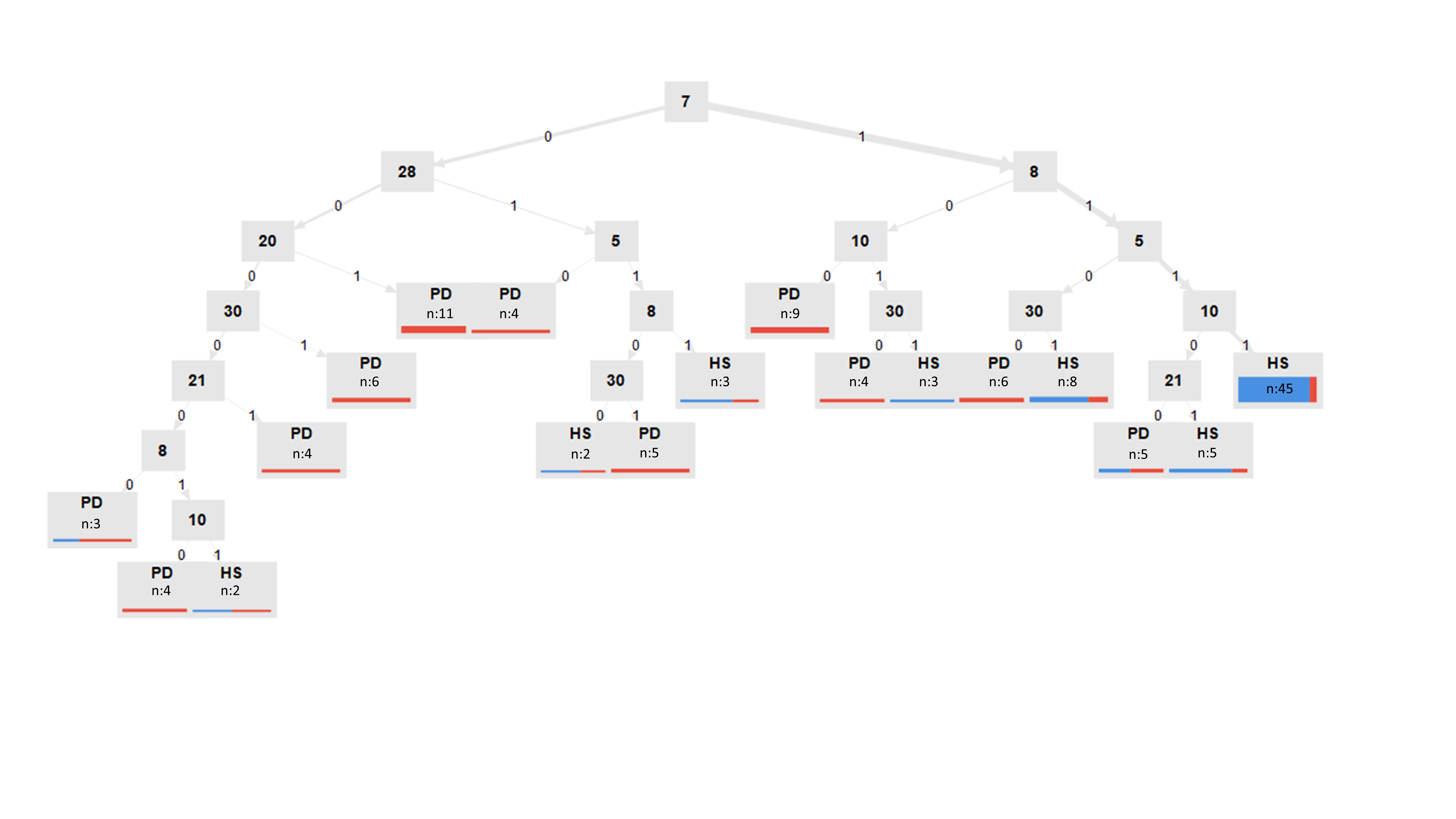

Supplement: Supplementary file 2 — High Resolution Image (TIF 2715 kb) [file 10072_2022_6457_MOESM1_ESM.tif]

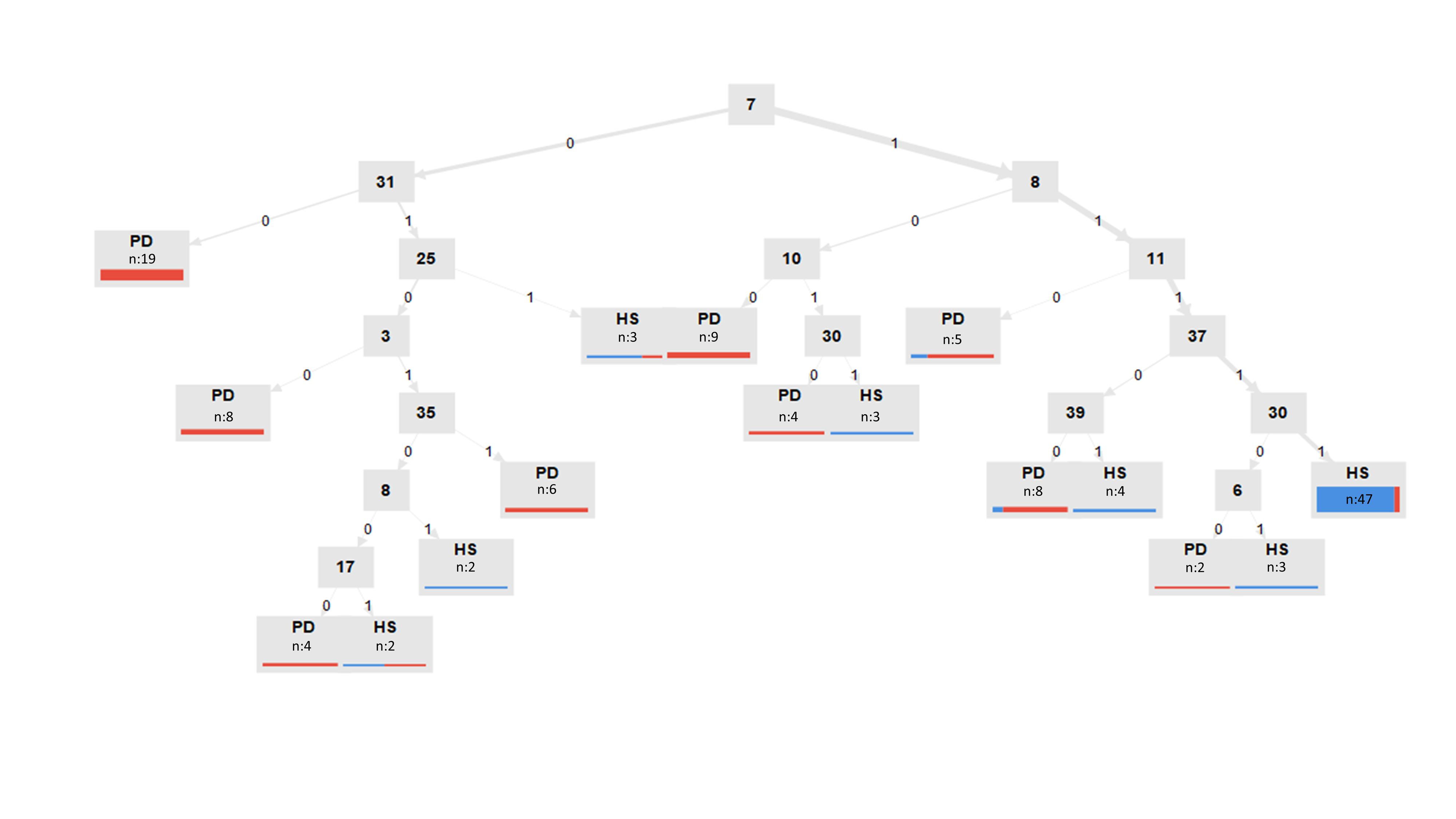

Supplement: Supplementary file 3 — Decision tree built with the whole UPSIT items. Grey boxes represent nodes defined by item (odor) number. Branches are defined by the given answer to each item (0=incorrect; 1=correct). Red bars represent PD patients; blue bars represent HS; bar thickness represents the number of patients defined by each node; this number is also shoved within each box as “n:” For each bar, the red and the blue relative width represents the percentage of PD patients and HS identified by each node. (PNG 337 kb) [file 10072_2022_6457_Fig4_ESM.png]

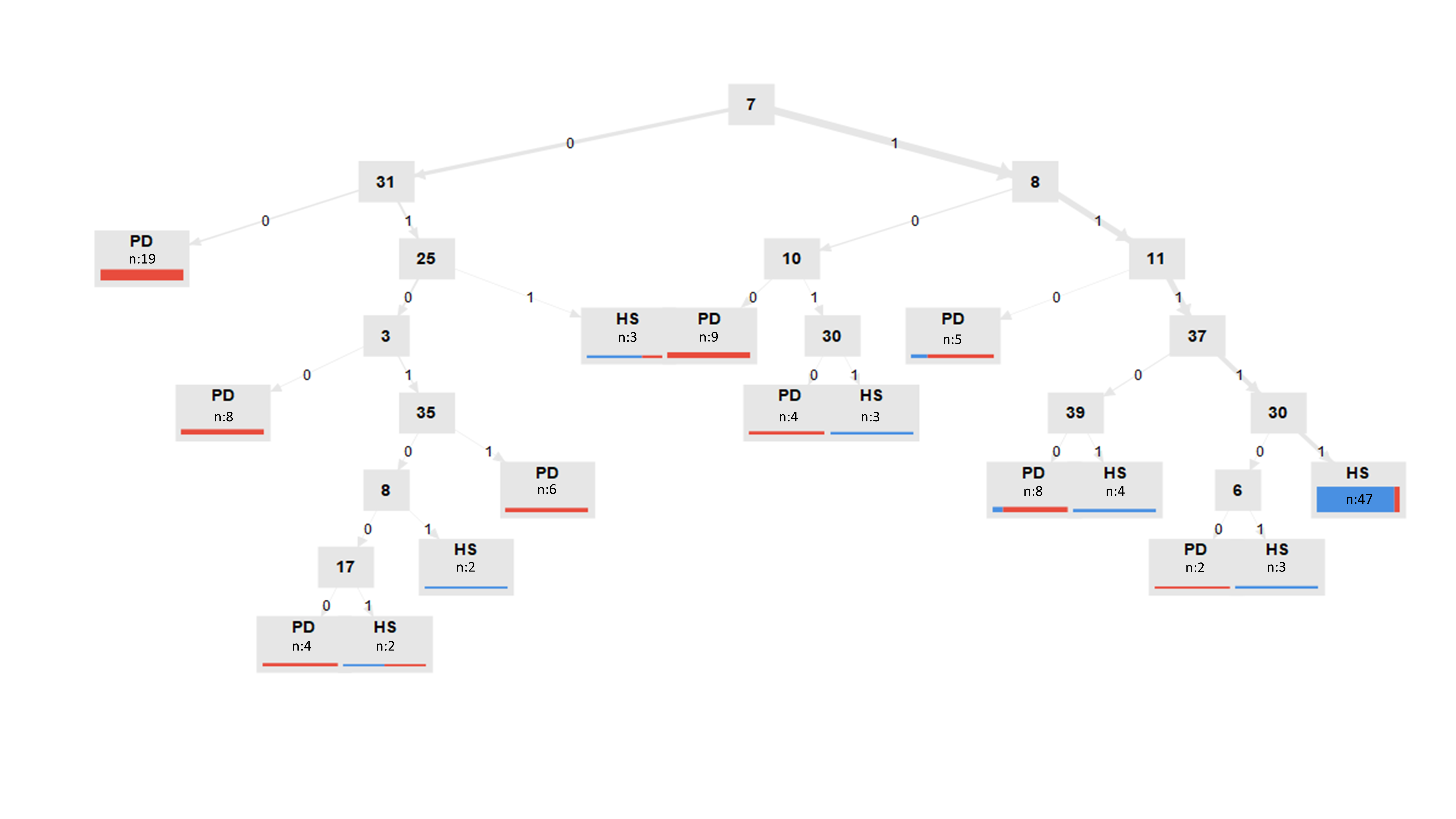

Supplement: Supplementary file 4 — High Resolution Image (TIF 2713 kb) [file 10072_2022_6457_MOESM2_ESM.tif]
