# Supplementary material for: Screening performances of an 8-item UPSIT Italian version in the diagnosis of Parkinson’s disease
Source: Neurol Sci. 2022 Nov 19;44(3):889–95. doi: 10.1007/s10072-022-06457-2 (PMC9676802; doi:10.1007/s10072-022-06457-2)
Supplement: Supplementary file 5 — (PDF 75 kb) [file 10072_2022_6457_MOESM3_ESM.pdf]

Screening performances of an 8-item UPSIT Italian version in the diagnosis of Parkinson's Disease

Annamaria Landolfi, MD[1], Marina Picillo, MD, PhD[1], Maria Teresa Pellecchia, MD, PhD[1], Jacopo Troisi, MSc, MBBS[2,3], Marianna Amboni, MD, PhD[1,4], Paolo Barone, MD, PhD[1], Roberto Erro, MD, PhD[1]

1. Department of Medicine, Surgery and Dentistry "Scuola Medica Salernitana", Neuroscience section, University of Salerno
2. Theoreo srl, Via degli Ulivi 3, 84090 Montecorvino Pugliano, Italy
3. Department of Chemistry and Biology "A.Zambelli", University of Salerno, Fisciano, Italy
4. Istituto di Diagnosi e Cura Hermitage-Capodimonte, Naples, Italy

Correspondence:

Annamaria Landolfi

Department of Medicine, Surgery and Dentistry "Scuola Medica Salernitana",

Neuroscience section, University of Salerno

Via Allende 43, 84081 Baronissi, (SA) Italy

anlandolfi@unisa.it; a.landolfi@hotmail.it

Supplementary information

Logistic regression

The Logistic regression (LR) model is part of the class of generalized linear models. It is a nonlinear regression model used when the dependent variable is dichotomous. The objective of the model is to establish the probability with which an observation can generate one or the other value of the dependent variable; it can also be used to classify observations, based on their characteristics, into two categories [1].

Linear discriminant analysis

Linear discriminant analysis (LDA) is employed to look for the linear combination of features which best separates the two classes. The resulting combination is used as a linear classifier. LDA is closely related to ANOVA (analysis of variance) and regression analysis, the aim of which is to express one dependent variable as a linear combination of other features or measurements. LDA is also closely related to principal component analysis (PCA) and factor analysis, since both look for linear combinations of variables which best explain the data. More precisely, LDA attempts to model the difference between the classes of data [2].

The Decision-Tree model

A decision tree is a tree-like collection of nodes intended to create a decision on value affiliation to a class. In our case, each node represents an UPSIT item, each branch a possible decision related to that node (in our case, correct vs. incorrect answer). Hierarchical selection of the items is based on information gain, which represents an

estimate of entropic reduction of the information initially contained in data by means of the choice that can be attributed to that item. Therefore, each node represents a splitting rule for one specific UPSIT item. The building of new nodes is repeated until the stopping criterion (i.e., the class) is met. A prediction for the class label attribute is determined depending on the majority of the cases which reached this leaf during generation [3].

Supplementary section references

1. Stock JH, Watson MW (2015) Regression with a Binary Dependent Variable. In: Introduction to Econometrics, III ed. Pearson
2. Xanthopoulos P, Pardalos PM, Trafalis TB (2013) Linear discriminant analysis. In: Robust data mining. Springer, pp 27–33
3. Breiman L, Friedman J, Stone CJ, Olshen RA (1984) Classification and regression trees. CRC press

Supplementary tables

Supplementary table 1a. Top 12 discriminating odors selected for each statistical method employed. Items are ordered from the best to the worst discriminating one. In bold, the items that, being selected by at least 4 out of 5 statistical models, represent the final item subset (LR=logistic regression; LDA=linear discriminant analysis; AUC-ROC= Area Under the Receiver Operating Characteristic Curve).

Fisher's χ^2	Odds Ratio	LR	LDA	AUC-ROC
**Banana**	Talc	Onion	**Banana**	**Banana**
**Clove**	Onion	Talc	**Clove**	**Clove**
**Watermelon**	**Banana**	**Lilac**	**Watermelon**	**Watermelon**
**Apple**	**Clove**	Leather	**Apple**	**Orange**
**Orange**	**Watermelon**	Chewing gum	**Orange**	**Apple**
**Lilac**	**Apple**	Chocolate	**Motor Oil**	**Lilac**
**Motor Oil**	Leather	**Coconut**	**Lilac**	Walnut
Walnut	**Motor Oil**	Grape	**Coconut**	**Coconut**
**Coconut**	Soap	Pine	Walnut	**Motor Oil**
Rose	**Orange**	Cinnamon	Talc	Rose
Soap	**Lilac**	**Apple**	Rose	Fruit Juice
Diluent	**Coconut**	**Orange**	Onion	Grape

Supplementary table 1b. Worst 12 discriminating odors as selected by each statistical method employed. (LR=logistic regression; LDA=linear discriminant analysis; AUC-ROC= Area Under the Receiver Operating Characteristic Curve).

Fisher's χ^2	Odds Ratio	LR	LDA	AUC-ROC
Mint	Cinnamon	Peanut	Mint	Mint
Menthol	Menthol	Strawberry	Menthol	Menthol
Liquorice	Liquorice	Clove	Liquorice	Liquorice
Lemon	Lemon	Pineapple	Lemon	Lemon
Cedar	Cedar	Mint	Cedar	Pine
Pine	Pine	Lemon	Pine	Cedar
Raspberry	Lime	Peach	Raspberry	Raspberry
Lime	Dill Pickle	Pizza	Lime	Lime
Chocolate	Smoke	Cedar	Chocolate	Chocolate
Dill Pickle	Grass	Raspberry	Dill Pickle	Dill Pickle
Grass	Chocolate	Cherry	Grass	Grass
Smoke	Raspberry	Dill Pickle	Smoke	Smoke
